# Constructing a novel prognostic model for triple-negative breast cancer based on genes associated with vasculogenic mimicry

**DOI:** 10.18632/aging.205806

**Published:** 2024-05-08

**Authors:** Yu Ren, Luyi Feng, Zhihua Tan, Fulin Zhou, Shu Liu

**Affiliations:** 1 Department of Clinical Medicine, Guizhou Medical University, Guiyang, China; 2 Department of Breast Surgery, The Affiliated Hospital of Guizhou Medical University, Guiyang, China; 3 Information Department of Guizhou Provincial People’s Hospital, Guiyang, China; 4 Department of Breast Surgery, Guiyang Maternal and Child Health Care Hospital, Guiyang, China; 5 The Maternal and Child Health Care Hospital of Guizhou Medical University, Guiyang, China

**Keywords:** triple negative breast cancer, vasculogenic mimicry, prognosis, biomarkers, function

## Abstract

Background: Research has shown a connection between vasculogenic mimicry (VM) and cancer progression. However, the functions of genes related to VM in the emergence and progression of TNBC have not been completely elucidated.

Methods: A survival risk model was constructed by screening biomarkers using DESeq2 and WGCNA based on public TNBC transcriptome data. Furthermore, gene set enrichment analysis was performed, and tumor microenvironment and drug sensitivity were analyzed. The selected biomarkers were validated via quantitative PCR detection, immunohistochemical staining, and protein detection in breast cancer cell lines. Biomarkers related to the proliferation and migration of TNBC cells were validated via *in vitro* experiments.

Results: The findings revealed that 235 target genes were connected to the complement and coagulation cascade pathways. The risk score was constructed using KCND2, NRP1, and VSTM4. The prognosis model using the risk score and pathological T stage yielded good validation results. The clinical risk of TNBC was associated with the angiogenesis signaling pathway, and the low-risk group exhibited better sensitivity to immunotherapy. Quantitative PCR and immunohistochemistry indicated that the expression levels of KCND2 in TNBC tissues were higher than those in adjacent nontumor tissues. In the TNBC cell line, the protein expression of KCND2 was increased. Knockdown of KCND2 and VSTM4 inhibited the proliferation and migration of TNBC cells *in vitro*.

Conclusions: In this study, three VM-related biomarkers were identified, including KCND2, NRP1, and VSTM4. These findings are likely to aid in deepening our understanding of the regulatory mechanism of VM in TNBC.

## INTRODUCTION

Worldwide, breast cancer is the leading cause of cancer-related deaths [1] and triple-negative breast cancer (TNBC) constitutes 15% of all breast cancer incidences [2]. TNBC displays high invasiveness, and its recurrence and metastasis rates surpass those of other breast cancer types [3, 4]. The absence of viable treatment targets in TNBC restricts treatment options to conventional methods, such as adjuvant and neoadjuvant chemotherapy, along with surgery and radiation therapy in certain patients.

Angiogenesis plays a substantial role in the development, progression and dissemination of highly invasive forms of cancer, and current antiangiogenic therapies targeting the vascular endothelial growth factor (VEGF) have not yielded satisfactory results [5–7]. Unlike angiogenesis, which involves endothelial cells, vasculogenic mimicry (VM) pertains to the formation of vascular structures directly by the cancerous cells. The initial discovery of VM was in the realm of melanoma [8], where melanoma cells form a three-dimensional, conduit-like vascular network. This process is referred to as “VM” and has been observed in several highly invasive tumors [9]. In breast cancer, VM is linked to the presence of highly malignant phenotypes, including triple-negative and HER2-positive neoplasms [10, 11]. VM can contribute to the progression of TNBC in multiple ways. The stem cell markers ALDH1 and CD133, which are highly expressed in TNBC [12], are implicated in the formation of VM and are linked to decreased overall survival rates and increased likelihood of metastasis in patients with breast cancer [13]. Furthermore, VM and previously mentioned stem cell markers participate in the epithelial-mesenchymal transition (EMT) aspects of TNBC cells. Owing to HIF1α factor activation, TWIST1 transcription is stimulated, which in turn increases the number of CD133 cells and promotes vessel development via VM [14]. Therefore, there is an urgent requirement related to discover prognostic biomarkers pertinent to VM in the context of TNBC.

Currently, extensive research is being conducted on prognostic models for TNBC. For instance, models predicting the prognosis of patients with TNBC post-surgery based on modes of cell death [15], prognostic models integrating mitochondrial RNA (mRNA)–large noncoding RNA via the transcriptomic analysis of TNBC [16], and prognostic models based on exosome-related and aging-related genes [17, 18] have been proposed. However, research on prognostic models related to angiogenic mimicry in TNBC has not been reported so far. In this study, we identified three biomarkers associated with VM were identified via bioinformatics analysis and constructed a novel prognostic model for TNBC was constructed. Gene set enrichment analysis (GSEA) revealed an association emerged between the clinical risk of TNBC and signaling pathways related to angiogenesis. The connection between the functionalities of high and low-risk groups and the tumor microenvironment (TME) and in addition to drug sensitivity within these diverse risk categories were investigated, offering potential clinical targets.

## MATERIALS AND METHODS

### Extracting information

The RNA sequencing data, as well as survival and clinical details pertaining to TNBC, were collected from The Cancer Genome Atlas (TCGA, accessible at https://portal.gdc.cancer.gov) and from the Gene Expression Omnibus (GEO, available at https://www.ncbi.nlm.nih.gov/geo/) databases. The TCGA-TNBC dataset comprises 116 TNBC and 113 healthy control (HC) samples, of which, 115 TNBC samples with survival information were randomly grouped in the ratio of 7:3. These samples were utilized as the training dataset and internal validation datasets, respectively. The GSE135565 was employed as the external validation dataset which permitted the confirmation of the survival risk model’s availability. This dataset encompasses 84 TNBC samples with complete with survival data. In addition, 24 vasculogenic mimicry-related genes (VMRGs) were procured from preceding studies (Supplementary Table 1) [19].

### Functional enrichment analysis of target genes

In this research, we contrasted differentially expressed genes (DEGs) from 116 TNBC and 113 HC samples were contrasted within the TCGA-TNBC dataset by employing the “DESeq2” R package (version 1.34.0) with |log2FC| > 1, adj.p.value < 0.05 [20]. Following this, a coexpression network was constructed using the “WGCNA” R package to isolate module genes linked to the score of VMRGs [21]. Subsequently, using the “Venn” function, we identified target genes at the intersection of DEGs and module genes were identified. Moreover, the “clusterProfiler” R package (version 4.2.2) was used for functional enrichment analysis of these target genes (adj.p.value < 0.05) [22].

### Building the survival risk model and forecast model related to TNBC

In this investigation, we gathered 81 TNBC samples collected from the TCGA dataset to construct the survival risk model, also referred to as the risk score. Biomarkers for TNBC were identified via univariate and multivariate Cox analyses. The risk score was computed using the following formula: Riskscore = β1X1 + β2X2 + ... + βnXn. To predict the precision of the survival risk model, methods such as the Kaplan-Meier (K-M) survival curve, risk curve, and receiver operating characteristic (ROC) curve were used. Moreover, to confirm the suitability of this model, both the internal validation dataset from TCGA and an external validation dataset, GSE135565, were used.

The clinical characteristics of TNBC included risk score, race, age, and stage, (pathologic T, pathologic N, pathologic M). Next, key prognostic variables identified via univariate and multivariate Cox analyses were employed to construct the prognostic model, also known as a nomogram. Subsequently, to assess the validity of the prognostic model, the calibration curve of the nomogram was plotted.

### Function and tumor micro-environment (TME) analyses in different risk groups

GSEA was performed to explore the functionality of genes in different risk categories using the R packages “clusterProfiler” and “org.Hs.eg.db” (adj.p.value < 0.05). In addition, to gain deeper insights into the canonical pathways of biomarkers, ingenuity pathway analysis (IPA) was performed [23].

Furthermore, using the “CIBERSORT” algorithm, the distribution of immune cells between various risk categories was quantified. The associations between immune cells and the connections between biomarkers and various immune cells were then calculated using “Spearman”. Moreover, the immunotherapy sensitivity in different risk groups was evaluated using the “SubMap” algorithm.

### Drug sensitivity analysis

The half maximal inhibitory concentration (IC_50_) of 138 common chemotherapeutic drugs for TNBC in different risk groups were compared using the “pRRophetic” R package (version 0.5) [24].

### Clinical sample

A total of 20 pairs of TNBC tissues and their corresponding adjacent cancerous tissues were collected. These came from patients who received treatment at the Affiliated Hospital of Guizhou Medical University. Moreover, to prepare these clinical samples for future use, they were subjected to a process of fixation with formalin and embedding in paraffin. For this study, ethical approval was obtained from the Ethics Review Committee of the First Affiliated Hospital of Guizhou Medical University. All participating patients provided their written informed consent in compliance with pertinent guidelines. The TNBC tissues and their corresponding peritumoral controls were promptly placed in liquid nitrogen and preserved at −80° C.

### Cell culture

The cell lines MDA-MB-231, BT-549, 578T, HCC1937, MCF7, SKBR3, BT-474 and MCF-10A were acquired from Procell Life Science and Technology (Wuhan, China). These cells were grown in a DMEM medium (C11995500BT, Gibco, USA). Each of the culture medium was supplemented with 10% fetal bovine serum (FBS, Z7185FBS-500, ZETA, USA). The cells were incubated at 37° C and 5% carbon dioxide.

### RT-qPCR

The total RNA within the tissues was extracted using the TRIzol method. (Thermo Fisher Scientific, USA). The Reverse Transcription Reagent Kit (Accurate Biology, Hunan, China) was then applied to transcribe mRNA into cDNA, which provided a template for the amplification of target genes in the ensuing qPCR experiments. The CFX96 Real-Time System (Bio-Rad, USA) served as the detection instrument for conducting quantitative real-time PCR. The following primer sequences (5’-3’) were used: KCND2 (forward; AGTAATCAGCTGCAGTCCTCAGA, reverse; TGTTCGTCCACAAACTCGTGA), VSTM4 (forward; CTTTGCACACTCCTTCGACTC, reverse; GACGTAATGCCCTTGATCGGA), β-action (forward; CTGGAACGGTGAAGGTGACA, reverse; AAGGGACTTCCTGTAACAATGCA).

### Western blotting and antibodies

The procedure of western blotting was performed as per standard techniques. Antibodies to KCND2 (21298-1-AP), were purchased from Proteintech (Wuhan, China). Anti-β-actin (AP0060) was procured from Bioworld (Nanjing, China). Mini Trans-Blot devices (Bio-Rad) were employed to perform western blotting. Subsequently, the bands were visualized and were captured using a chemiluminescence imaging system (Minichemi).

### Immunohistochemistry (IHC)

Paraffin-embedded tissue samples from clinical cases of TNBC were cut into 4-μm thick slices. Subsequently, these slices were deparaffinized with xylol, rehydrated using graduated alcohol, and subjected to antigen retrieval by heating. Endogenous peroxidase activity was eliminated using 3% H_2_O_2_, and the sections were incubated with KCND2 (21298-1-AP, Proteintech) at 4° C for an overnight duration. After 30-min open incubation with secondary antibodies, the sections were visualized and documented with the Olympus microscope (BX53 M, Olympus, Japan).

### Transfection

The siRNAs for KCND2 and VSTM4 were designed and synthesized by RiboBio (Guangzhou, China). KCND2 siRNA1: CCTGGAACGTTACCCAGACACTCTA; KCND2 siRNA2: AAGCTGCATGGAAGTTGCAACTGTT; KCND2 siRNA3: ACAACCTTATGTGACTACAGCAATA. VSTM4 siRNA1: TAACCTATGCCGAACTGGAGCTGAT; VSTM4 siRNA2: CCACGGAAATGAGAGTCATTTCCCT; VSTM4 siRNA3: CCTTGTAATACTACCTCACTCTTTG. MDA-MB-231 and BT-549 cells were seeded in 6-well plates at a confluency of 40–60% and then transfected with Lipofectamine 3000 (Invitrogen Biotechnology, USA) for 24–36 h. The cells were then collected for further experiments.

### EdU incorporation assay

The transfected cells were seeded in 96-well plates at a density of 8,000 cells per well, and experiments were conducted in triplicate for each condition in all groups. Twelve hours post-seeding, the medium was replaced with serum-free medium and cultured for 24 h to synchronize the cell cycle. The Cell-Light EdU Apollo 567 *In Vitro* Kit (RiboBio, Guangdong, China) was used according to the manufacturer’s instructions.

### Cell counting kit 8 assay

Cells were seeded at a density of 3,000 cells per well in 96-well plates, with quadruplicate repeats for each condition. A Cell Counting Kit-8 (CCK8, Beyotime Biotechnology, Shanghai, China) was added every 24 h for 4 days. Two hours after the CCK8 treatment, the optical density value of each well was measured at 450 nm.

### Wound healing assays

After the transfection of the breast cancer cells, they were cultured in six-well plates until confluence. A 10-μL pipette tip was used to vertically scratch the cells, and the scratched cells were washed with phosphate-buffered saline. Subsequently, 2 mL of 1.25% FBS medium was added, and the culturing of the cells was continued for 24 h. Finally, the cells were observed to calculate the average wound gap between the edges of the wound.

### Statistical analysis

Calculations were performed using the R programming language (https://www.r-project.org/). For survival correlation assessment, methods included K-M curves, log-rank tests, and univariate and multivariate analyses with the Cox proportional hazards approach were used. Categorical variables were evaluated using the student’s t-test. To examine the relationship between two variables, Wilcoxon correlation testing was used. In this study, a P-value of<0.05 indicated statistical significance.

### Data availability statement

The data presented in this study are openly available. The gene expression data, patient clinical information, and patient survival information of the TCGA-TNBC cohort were downloaded from the TCGA database (https://portal.gdc.cancer.gov/). The external validation set was the TNBC-related GSE135565 dataset downloaded from the GEO database (https://www.ncbi.nlm.nih.gov/geo/).

## RESULTS

### Target genes were associated with complement and coagulation cascades

**Figure 1:**
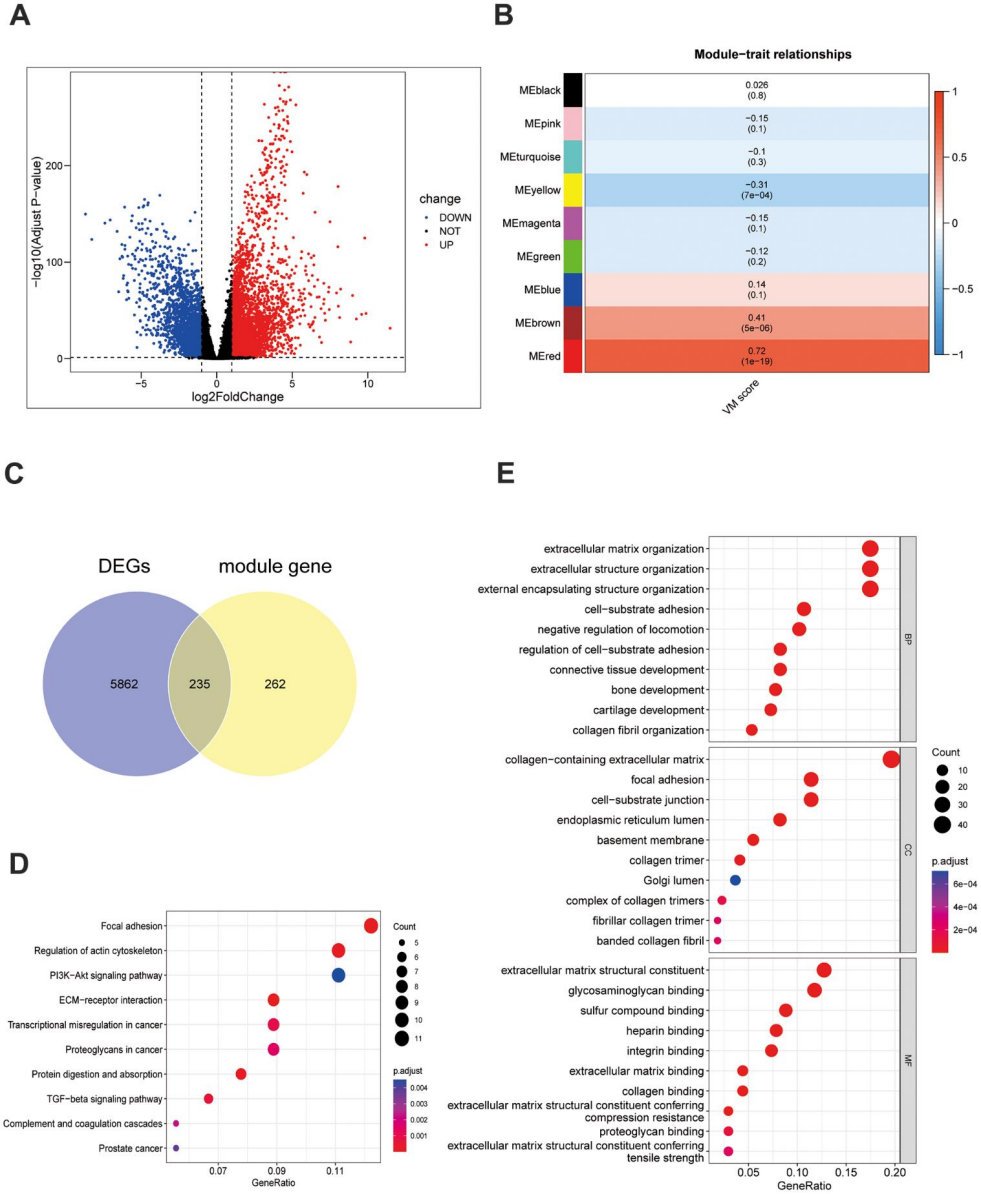
**Screening and function of VM-related genes in TNBC.** (**A**) Differentially expressed genes in the TCGA-TNBC dataset. (**B**) The VM scores are associated with 9 gene modules. These results were then visualized in a heatmap. (**C**) The Venn diagram depicts 235 differentially expressed VM-associated genes. (**D**) The 235 differentially expressed genes associated with VM underwent a KEGG functional enrichment analysis. (**E**) GO analysis was conducted on the 235 differentially expressed VM-associated genes.

In the TCGA-TNBC dataset, comparing 116 TNBC samples with 113 HC samples revealed the presence of 6,097DEGs of these, 3,704 genes were upregulated and 2,393 were downregulated (Figure 1A). A sample clustering analysis was performed, the results of which indicated a single outlier sample which was consequently excluded from further analyses (Supplementary Figure 1A). When identifying the optimal soft threshold value, a value of six brought the network close to a scale-free distribution. Using a hybrid dynamic tree-cutting algorithm, nine modules were extracted (Supplementary Figure 1B, 1C). The correlation results demonstrated that the MEred module had a significantly positive correlation with the scores of VMRGs (cor = 0.72, *p* = 1e-19) (Figure 1B). Hence, this module was pinpointed as the critical module and 497 module genes were selected for subsequent analysis. An intersection between the 6,097 DEGs and 497 module genes resulted in a total of 235 target genes (Figure 1C and Supplementary Table 2). In terms of functionality, these targets were predominantly enriched in the processes of fostering cell-substrate adhesion positively, impeding cell migration via negative regulation, promoting epithelial cell proliferation, migration, transmembrane receptor protein serine/threonine kinase signaling pathways etc., which encompassed a total of 272 Gene Ontology (GO) functions in total. Furthermore, these targets were implicated in extracellular matrix (ECM)-receptor interaction, focal adhesion, complement coagulation cascades, and signalling pathways involving TGF-beta, P13K-Akt, Wnt, etc., which amounted to a total of 16 Kyoto Encyclopedia of Genes and Genomes (KEGG) pathways (Figure 1D, 1E and Supplementary Tables 3, 4).

### KCND2, NRP1, and VSTM4 were used to construct the survival risk and prognostic models of TNBC

**Figure 2:**
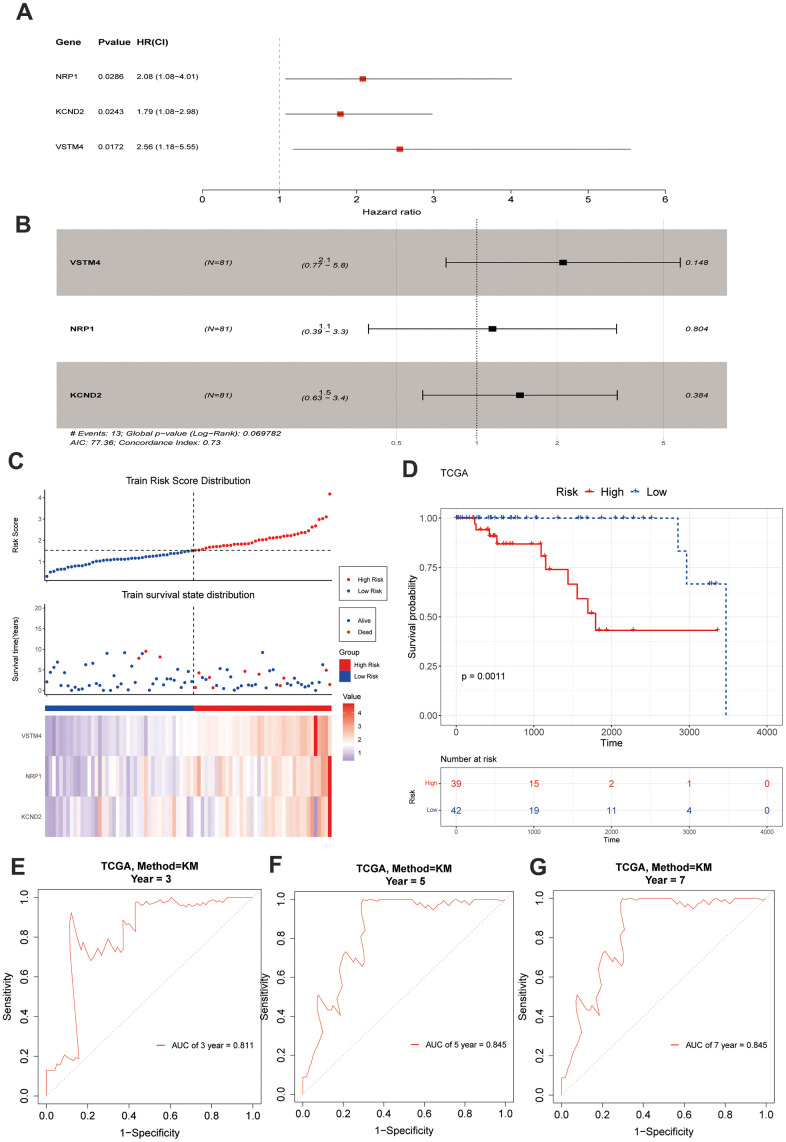
**Construction and evaluation of risk score.** (**A**, **B**) A forest chart exhibits VM-associated genes obtained through regression analysis. (**C**) The model delineates the patients' risk score distribution, their survival status, and a heatmap illustrating the gene expression. (**D**) A significant survival discrepancy was noted between the high-risk and low-risk groups, as evidenced by their respective KM survival curves. (**E**–**G**) The ROC curves from the training set display distinct AUC values corresponding to the 3-year, 5-year, and 7-year overall survival times, respectively.

**Figure 3:**
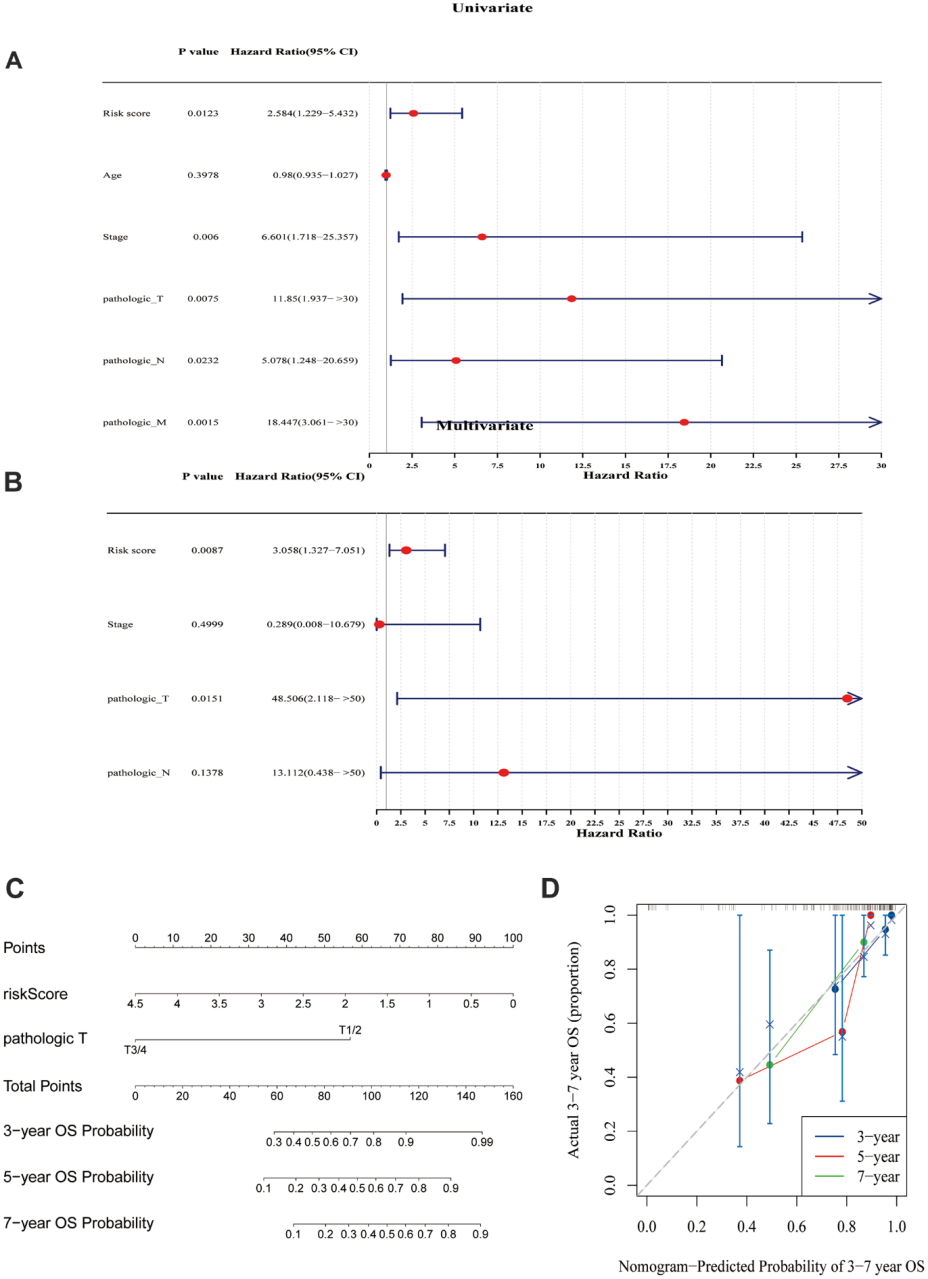
**Prognostic model analysis.** (**A**) Univariate Cox regression analysis was performed on the seven variables. (**B**) A Cox model was constructed using four variables. (**C**) A model was created and a nomogram was plotted, incorporating the risk scores derived from the multivariate Cox analysis and tumor staging. (**D**) Calibration curves were used to evaluate the prognostic model. The results suggested that the nomogram may be an effective tool for predicting the survival outcomes of TNBC patients.

Based on the above 235 target genes, three biomarkers, including KCND2, NRP1, and VSTM4, were identified, all of which were found to be negative factors (hazard ratio > 1) of TNBC (Figure 2A, 2B). The risk curve, in conjunction with the K–M curve, demonstrated significant survival disparities between the two risk groups (p-value = 0.0011). (Figure 2C, 2D). Furthermore, the areas under the ROC curves (AUC values) of 3-, 5-, and 7-year were >0.8 (Figure 2E–2G). In addition, the internal and external datasets were used to verify the applicability of this survival risk model, respectively. The results of the risk curve, K–M curve, and ROC curve were consistent with the training dataset (Supplementary Figure 2A–2J). These findings indicated that this survival risk model could be used to construct the prognostic model of TNBC. On this basis, two factors—pathologic T and risk score—were identified to be linked to the prognosis of TNBC, and both negatively impacted patient survival (hazard ratio > 1) (Figure 3A, 3B). A nomogram incorporating these two prognostic indicators was built. The calibration curve illustrated that the slopes for the 3-, 5-, and 7-year survival rates approached 1. This observation implies that the nomogram could serve as an efficacious prognostic model for TNBC (Figure 3C, 3D).

### The clinical risk of TNBC was associated with angiogenesis-related signaling pathways

**Figure 4:**
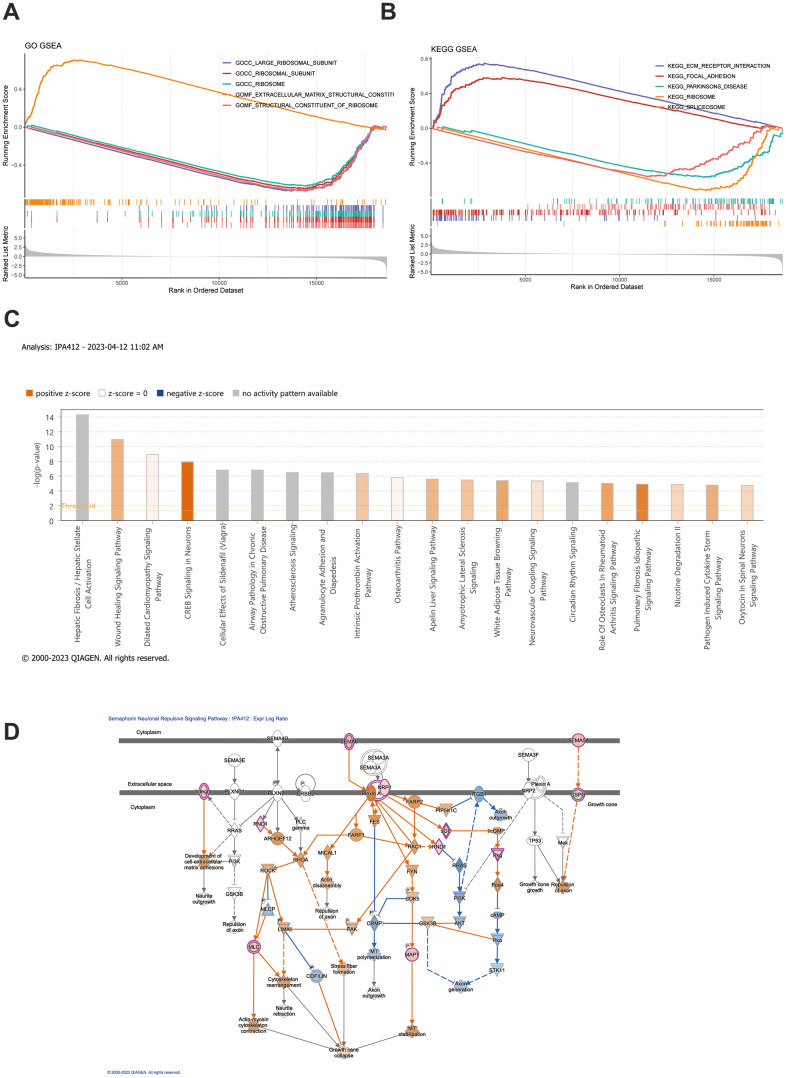
**GSEA and IPA analysis.** (**A**, **B**) GSEA enrichment analysis was conducted based on both GO gene sets and KEGG gene sets. The top 5 most significant pathways were selected for depiction in the GSEA enrichment analysis. (**C**) IPA examination uncovers the abundance of distinct gene expression between the high-risk and low-risk groups. (**D**) Depiction of the functional roles of the key mRNAs in the signaling pathway corresponds to the highest absolute z-score.

The GSEA results signified that the GO functions of platelet-derived growth factor binding, a protein complex involved in cell adhesion, and etc., were highly enriched in the high-risk group, and the functions of mitochondrial gene expression, oxidative phosphorylation, and etc., were significantly highly enriched in the low-risk group (Figure 4A and Supplementary Table 5). Moreover, the ECM receptor interaction, focal adhesion, vascular smooth muscle contraction, TGF-β signaling pathway (KEGG pathways), etc., were highly enriched in high-risk group, and NOD-like receptor signaling pathway, cell cycle, oxidative phosphorylation, etc., were significantly highly enriched in low-risk group Figure 4B and Supplementary Table 6). Moreover, IPA results indicated that these biomarkers were linked to four classical pathways, which included guidance signaling, semaphorin neuronal repulsive signaling pathway, semaphorin signaling in neurons, and VEGF family ligand-receptor interactions. Importantly, VEGF family proteins promoted vascular endothelial cell proliferation, migration, invasion, and angiogenesis (Figure 4C, 4D and Supplementary Table 7).

### The immunotherapy sensitivity was better in the low-risk group

**Figure 5:**
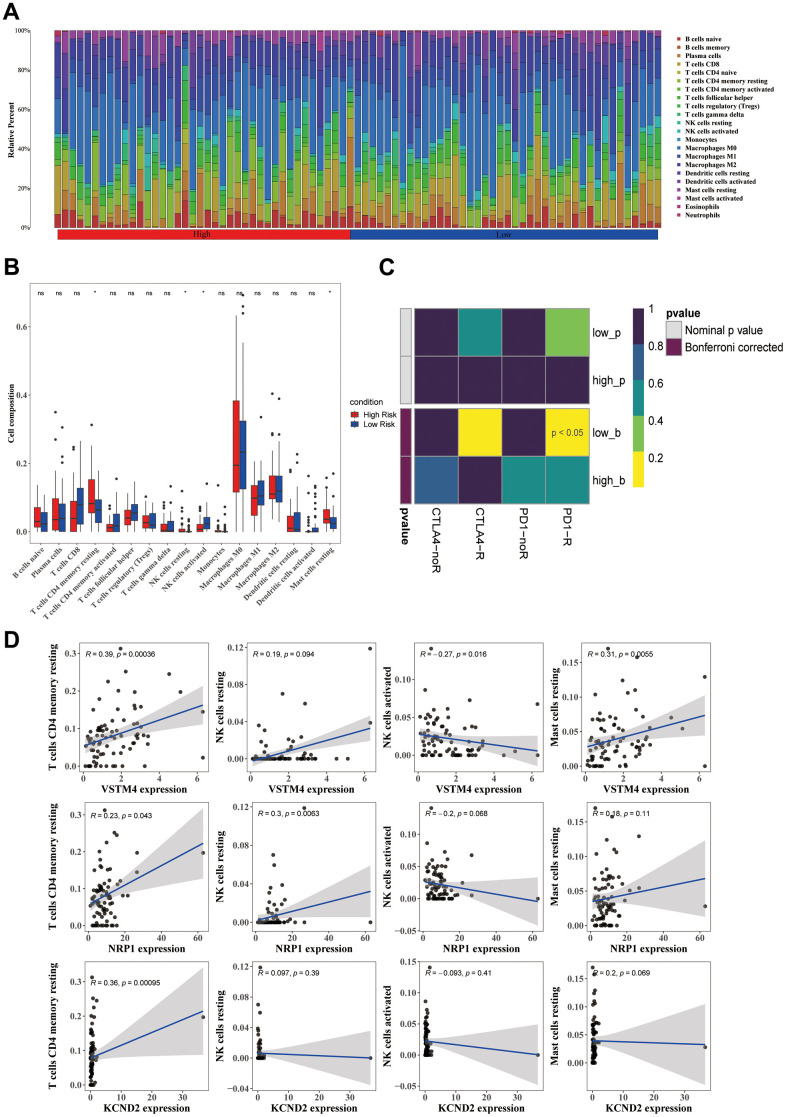
**Differences in the immune cells across various groups.** (**A**) The distribution proportion of immune cell abundance across all samples. (**B**) Differences in the abundance of immune cells between the high-risk and low-risk groups. (**C**) The correlation between prognostic genes and immune cells displays significant differences between the high-risk and low-risk groups. (**D**) Employing the SubMap method allowed an indirect estimation of the responsiveness to PD-1 and CTLA-4 immunotherapies in the high-risk and low-risk patient clusters.

Immune cells, such as resting mast cells, resting memory CD4 T cells, and resting natural killer (NK) cells, were significantly increased, whereas one immune cell (activated NK cells) was significantly decreased in the high-risk group (*p* < 0.05) (Figure 5A, 5B). Three biomarkers were positively correlated with the three immune cells that were significantly increased immune cells, and were negatively correlated with activated NK cells, which were significantly decreased, and the correlation results were consistent with their expressions. In addition, a significant positive correlation was observed between VSTM4 and resting memory CD4 T cells (R = 0.39, *p* = 0.00036) (Figure 5C). Furthermore, the group at a lower risk exhibited a significant immune response to PD-1 (*p* < 0.05), which suggested that sensitivity to immunotherapy was better in the low-risk group (Figure 5D). Moreover, the samples in the high-risk group were more sensitive to 25 drugs, including bicalutamide, dasatinib, and lapatinib, and the samples in the low-risk group were sensitive to 45 drugs, including axitinib, bosutinib, and gefitinib (*p* < 0.05) (Supplementary Figure 3A, 3B).

### Verification of KCND2 and VSTM4 in TNBC

**Figure 6:**
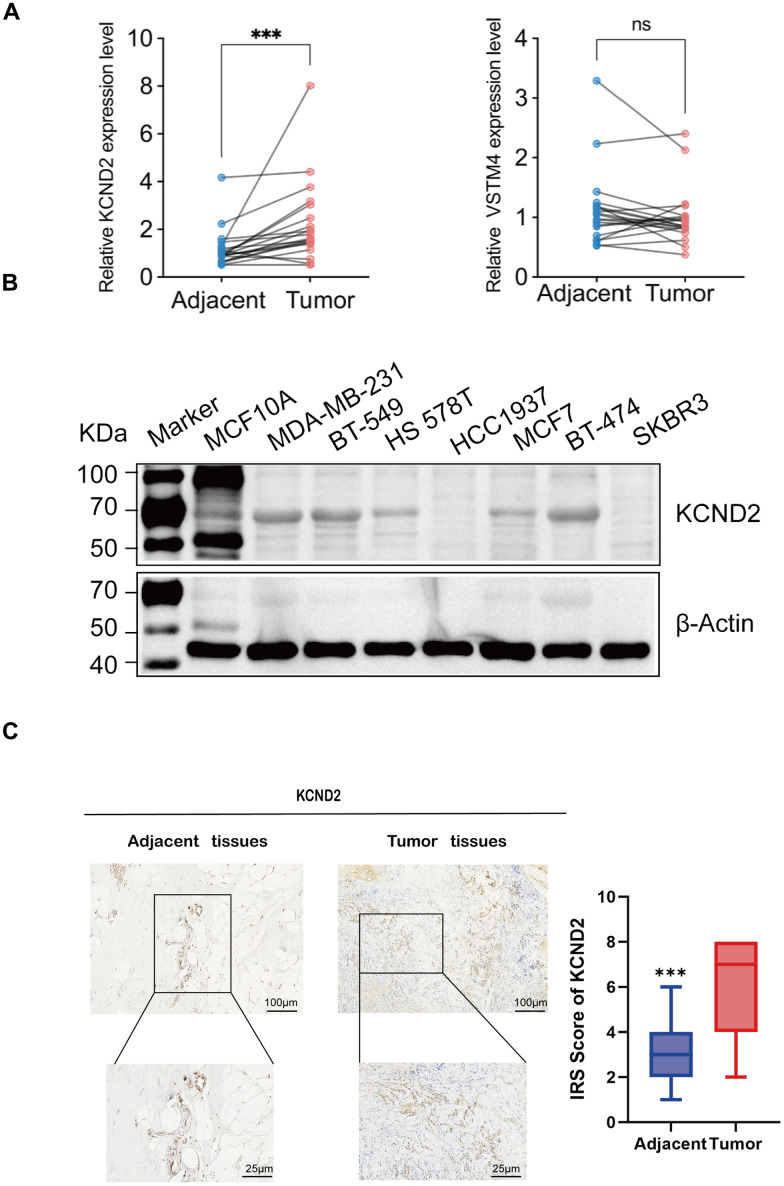
**Validation of biomarkers.** (**A**) The mRNA expression of KCND2 and VSTM4 in 20 pairs of tissues, including TNBC adjacent cancer and tumor tissues. (**B**) The protein expression of KCND2 was verified in cell lines. (**C**) The expression of KCND2 in the TNBC adjacent cancer and tumor tissues was verified by immunohistochemistry. NS, no significance; ***P < 0.001.

The qRT-PCR method was used to ascertain the expression levels of KCND2 and VSTM4 in 20 matching sets of TNBC and noncancerous breast tissues. The findings indicated that compared with adjacent noncancerous tissues, in TNBC tissues, there was a notable elevation in the expression of both KCND2, whereas VSTM4 was elevated in certain patients with TNBC (Figure 6A). The protein expression levels of KCND2 in various breast cancer cell lines were examined, which revealed that the expression was elevated in TNBC compared with normal cells or other breast cancer cell types (Figure 6B). Lastly, immunohistochemistry assays were performed for KCND2, which demonstrated that the expression of KCND2 in 20 pairs of TNBC samples was higher than that in matched adjacent samples (Figure 6C).

### Knockdown of KCND2 and VSTM4 prevented the proliferation and migration capabilities of TNBC cells

**Figure 7:**
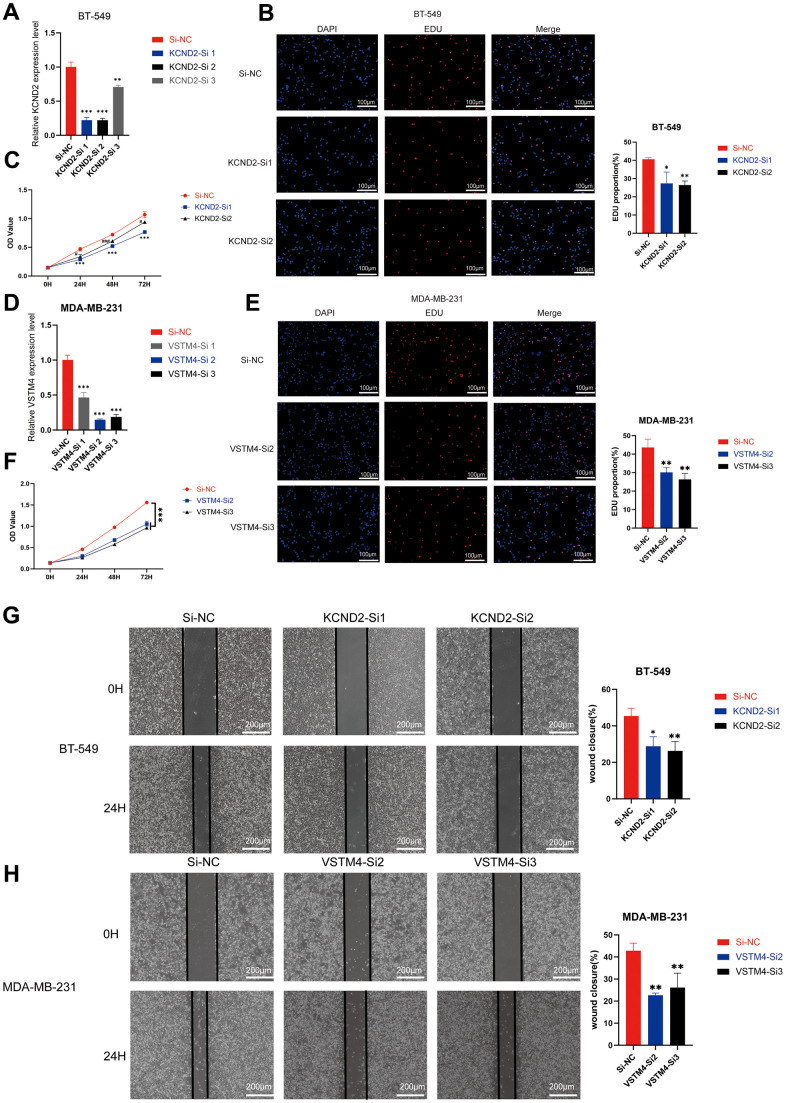
**Depletion of KCND2 and VSTM4 reduces the proliferation and migration of TNBC cells *in vitro*.** (**A**) The knockdown efficiency of siRNA targeting KCND2 in BT-549 cells was verified. (**B**) EdU incorporation analysis and (**C**) CCK-8 assay revealed that the knockdown of KCND2 affected the cell proliferation in BT-549 cells. (**D**) The knockdown efficiency of siRNA targeting VSTM4 in MDA-MB-231 cells was verified. (**E**) EdU incorporation analysis and (**F**) CCK-8 assay revealed that the knockdown of VSTM4 impacts cell proliferation in MDA-MB-231 cells. (**G**) The wound healing assay demonstrated that the knockdown of KCND2 affected the migration of BT-549 cells. (**H**) Wound healing assay results showed that the knockdown of VSTM4 impacted the migration of MDA-MB-231 cells. *P < 0.05, **P < 0.01, ***P < 0.001.

Small interfering RNA targeting KCND2 and VSTM4 were transfected into MDA-MB-231 and BT-549 cells, respectively, and the efficiency of the fragments was verified. Effective fragments were selected for subsequent experiments (Figure 7A, 7D and Supplementary Figure 4A, 4D). EDU and CCK-8 assays revealed that the knockdown of KCND2 and VSTM4 reduced the proliferation capability of TNBC cells (Figure 7B, 7C, 7E, 7F and Supplementary Figure 4B, 4C, 4E, 4F). Wound healing assays confirmed that the knockdown of KCND2 and VSTM4 decreased the migration ability of TNBC cells (Figure 7G, 7H and Supplementary Figure 4G, 4H).

## DISCUSSION

The growth process of malignant tumors requires a considerable amount of oxygen to sustain their continued proliferation. When the tumor size exceeds 2 mm, the mass is unable to acquire sufficient oxygen and faces with a hypoxic environment [25]. Therefore, the tumor necessitates the formation of new vessels need to be formed for oxygenation. VM, a method of vasculature formation noted in various malignant tumors in recent years, has not been completely understood. However, the study of the tumor growth substrate or “microenvironment” has implied an inseparable connection between the hypoxic environment during solid tumor growth and VM.

Characteristics linked to unfavorable prognosis in TNBC include increased rates of recurrence, axillary lymph node metastasis, reduced survival rates, increased tumor dimensions, and inferior histological grading [26, 27]. Presently, in breast cancer, a key limitation in the treatment against angiogenesis for breast cancer is that although it hinders angiogenesis, it could potentially foster the development of VM via the induction of hypoxia. This could potentially favor VM, which, in turn, may enhance distant metastasis [28, 29]. Hence, a study has proposed that the optimal strategy for inhibiting the formation of tumor vasculature is to concurrently suppress angiogenesis and VM [30]. In this study, 235 target genes linked to ECM-receptor interaction, focal adhesion, complement and coagulation cascades, TGF-β, PI3K-Akt, and Wnt signaling pathways were identified. These functionalities and pathways bear a close relationship to VM. An intricate association exists between thrombin expression in tumors and VM formation, as demonstrated by elevated thrombin levels in tumor samples from patients with VM [31]. Under the influence of hypoxia, the ECM is extensively degraded by matrix metalloproteinases (MMPs) produced by cancer cells, as a result, the resistance to alterations in cellular morphology throughout the process of VM is diminished and VM is promoted via facilitation of epithelial to endothelial transition. This mechanism is triggered by TGF-β [32], PI3K-Akt [33], and the Wnt/β-catenin pathway [34] and involves molecules such as ZEB1 [35], Twist1 [36], MMP-3 [37], and MMP-14 [38], which corroborates our findings. A prognostic risk score related to TNBC was constructed using the aforementioned 235DEGs associated with VM. The analysis suggested that NRP1, KCND2, and VSTM4 were associated with TNBC prognosis. NRP1 is a single-pass transmembrane glycoprotein that comprises extracellular, transmembrane, and intracellular domains. In previous studies, the high expression of NRP1, together with the occurrence of VM, has been linked to unfavorable outcomes in patients with TNBC as it promotes tumor proliferation and metastasis. Downregulation of NRP1 in TNBC also resulted in the reduced expression of VM-associated genes, such as ZEB1 and Twist1. NRP1 can drive tumor progression by activating the RAS-MAPK pathway via EGFR and PDGFR. In the presence of TGF-β1, NRP1, and TβRI are cointernalized, which enhances classical Smad2/3 signal transduction. VEGF can influence the autocrine survival of TNBC cells via NRP1 [39–41]. KCND2, which is a part of the potassium voltage-gated channel subfamily, has been recently reported to promote gastric cancer [42] and lung adenocarcinoma [43]. KCND2 has been earmarked by scientists as a crucial determinant in the prognosis of individuals with breast cancer. Its increased expression has been linked to a reduced survival period in these patients. KCND2 has been identified to be a factor that promotes invasiveness and metastasis potential in breast cancer. Furthermore, it regulates cell cycle fluctuations in breast cancer cells, thereby facilitating the progression from the G1 to the S phase [44]. VSTM4, which comprises 320 amino acids, is a transmembrane protein whose biological functions are akin to those of VEGF [45]. A study has indicated its high expression in patients with acute myeloid leukemia [46]. Furthermore, high levels of VSTM4 have been noted in the tumors of murine models of colon cancer and melanoma [47]. In breast cancer, short hairpin RNA screening and sequencing for tamoxifen resistance or sensitivity in patients with ER+ breast cancer patients revealed that the downregulation of VSTM4 can augment the sensitivity to tamoxifen treatment [48]. Our study has validated the upregulation of the abovementioned genes in TNBC via qRT-PCR of clinical specimens, IHC, and western blotting of cells. Cox regression indicated that the three VM-associated genes, namely, NRP1, KCND2, and VSTM4, can independently predict the prognosis of TNBC. Concurrently, a clinical prognostic model for patient survival prediction, was constructed by integrating the risk score with clinical indicators. The nomogram’s ability to predict survival at 3, 5, and 7 years demonstrated its effectiveness in forecasting the survival outcomes for patients with TNBC. The results of GSEA and IPA analysis were in line with prior findings, such as the significant enrichment of platelet-derived growth factor binding, proteins involved in cell adhesion complexes, ECM-receptor interaction, focal adhesion, and TGF signaling pathway in the high-risk group. Similarly, IPA results suggested a high degree of enrichment in the interaction of the VEGF family ligand-receptor in the pathways linked to prognostic genes between the high and low-risk groups. VEGF family proteins are associated with the promotion of proliferation, survival, migration, and invasion of vascular endothelial cells, angiogenesis, and increased vascular permeability [49]. In the TME, different immune cells either promote or inhibit cancer [50]. Our findings have shed light on the relationship between high and low-risk groups in the model and immune cells. This result supports the current research finding that resting memory CD4 T-cells are found in later-stage TNBC [51]. Moreover, resting mast cells are upregulated in high-risk TNBC populations [52]. The increase in activated NK cells can improve the efficacy of trastuzumab treatment for TNBC [53]. In addition, the association between the three screened biomarkers and the immune cells was comparable between the high and low-risk groups. Mast cells produce copious amounts of molecules that promote angiogenesis and lymphangiogenesis. Tumor-associated mast cells can alter multiple facets of cancer, such as angiogenesis, proliferation, immune regulation, and tissue remodeling. The experimental findings confirmed a reduction in tumor formation and growth in mast cell-deficient mutant mice which emphasizes the key role of mast cells play in tumor development. Moreover, human mast cells have been reported to express VEGF receptors 1 (VEGFR-1) and 2 (VEGFR-2), co-receptor (NRP1), and -2 (NRP2) receptors [54]. The anomalous expression of K+ channels in cancer cells is also involved in oncogenic signal transmission, and makes the transformed cells self-reliant with respect to survival and growth factors. In addition, it triggers metabolic alterations, sustains the stem-cell phenotype, stimulates tissue infiltration and metastasis, or increases treatment [55]. The cytotoxicity of activated NK cells is not only linked to intrinsic cellular activity and membrane changes but also associated with the activity of K+ ion channels [56]. Immune cells related to antitumor activities, such as CD8+ T cells [57], decrease with the increase in KCND2 expression. The fusion protein of VSTM4 and Fc (VSTM4-Fc) has previously been shown to inhibit the activation of human T cells. In the presence of T cell receptor signal transduction, VSTM4 substantially inhibits human T cell proliferation and markedly decreases the production of cytokines, such as IFN-γ, IL-2, and IL-17, cytokines in human T cells. VSTM4-Fc binds strongly to activated human T cells, whereas it binds weakly to resting, nonactivated T cells. Our research has indirectly confirmed these findings [58]. Moreover, drug sensitivity analyses were performed for the high and low-risk groups. Our finding revealed that the group identified as high-risk group exhibited heightened sensitivity to medications such as bicalutamide, dasatinib, and lapatinib. Of these, bicalutamide can reverse the decrease in E-cadherin in TNBC, thereby reversing EMT [59], which is associated with VM formation [60]. In TNBC, using functionalized vincristine plus dasatinib liposomes which have been tailored using the targeted molecule DSPE-PEG 2000 -c(RGDyK), both *in vivo* and *in vitro* can result in the nondetection of recognized VM channel markers. These include vascular endothelial cadherin (VE-Cad), focal adhesion kinase (FAK), PI3K, and MMP-2, and MMP-9. Consequently, the blood circulation time is prolonged, its accumulation in tumor tissues is amplified, treatment outcomes are enhanced, and VM channels are absent in mice afflicted with TNBC [61]. Studies have reported that anti-EGFR can inhibit VM and angiogenesis, thus reducing TNBC metastasis [62]. Furthermore, preclinical studies have stated that the EGFR inhibitor lapatinib exerts antiproliferative effects in TNBC [63]. Nonetheless, its clinical performance has been subpar [64], which may be related to the complex heterogeneity of TNBC. The lower-risk group samples were further found to show higher sensitivity to drugs such as axitinib, bosutinib, and gefitinib. Certain basic and early clinical trials have been conducted on these drugs in TNBC [65–68], but further foundational theories and larger clinical sample sizes are still required.

In this study, bioinformatics was used to screen for three biological markers with prognostic value, which are associated with VM in TNBC, from public databases. However, owing to issues with the accessibility of antibodies, the expression of VSTM4 was not examined via IHC and western blotting. Furthermore, the fact that the data were sourced from public databases may present certain limitations. Given the limited number of TNBC datasets in public databases and the poor validation performance of the available datasets, a 7:3 split was employed.

Considering the aforementioned limitations, further analysis is required to comprehend the functions of these biomarkers in TNBC, their upstream and downstream regulatory mechanisms, and their relationship with VM.

## CONCLUSIONS

In summary, our study identified biomarkers in TNBC related to VM with prognostic value by constructing a VM-risk scoring for TNBC. Both training and validation cohorts exhibited satisfactory predictive performance, which facilitated the prediction of outcomes for patients with TNBC. By performing multifactor Cox regression analysis on tumor staging and risk scores, a prognostic model was built and evaluated using calibration curves. The results showed that the model performed well and could be used for the clinical prediction of patients’ survival. Additionally, VM-related genes were found to be possibly associated with the immune microenvironment of TNBC, and drug sensitivity prediction was performed. These findings provide valuable references for the treatment of TNBC.

## Supplementary Material

Supplementary Figures

Supplementary Table 1

Supplementary Table 2

Supplementary Table 3

Supplementary Table 4

Supplementary Table 5

Supplementary Table 6

Supplementary Table 7
