# Supplementary material for: Constructing a novel prognostic model for triple-negative breast cancer based on genes associated with vasculogenic mimicry
Source: Aging (Albany NY). 2024 May 8;16(9):8086–109. doi: 10.18632/aging.205806 (PMC11132006; doi:10.18632/aging.205806)
Supplement: Supplementary Figures [file aging-16-205806-s001.pdf]

SUPPLEMENTARY FIGURES

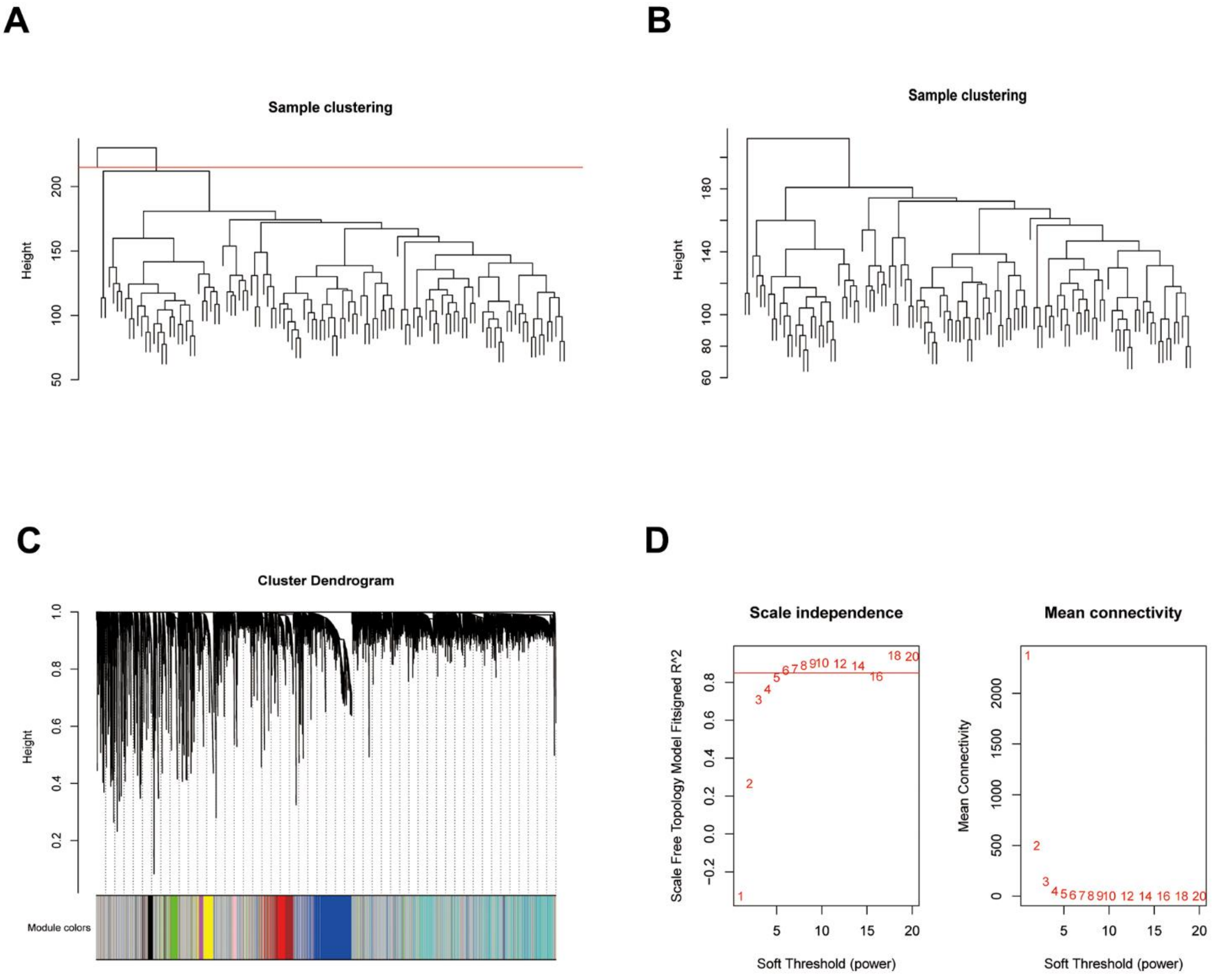

Supplementary Figure 1. Gene co-expression network examination with weighted approach. (**A**, **B**) A hierarchical clustering tree was built for all TNBC samples (N = 116), and one outlier sample was identified. This outlier was removed, and subsequent analyses were performed with the remaining samples (N = 115). (**C**) Hierarchical clustering results. (**D**) Soft-thresholding selection.

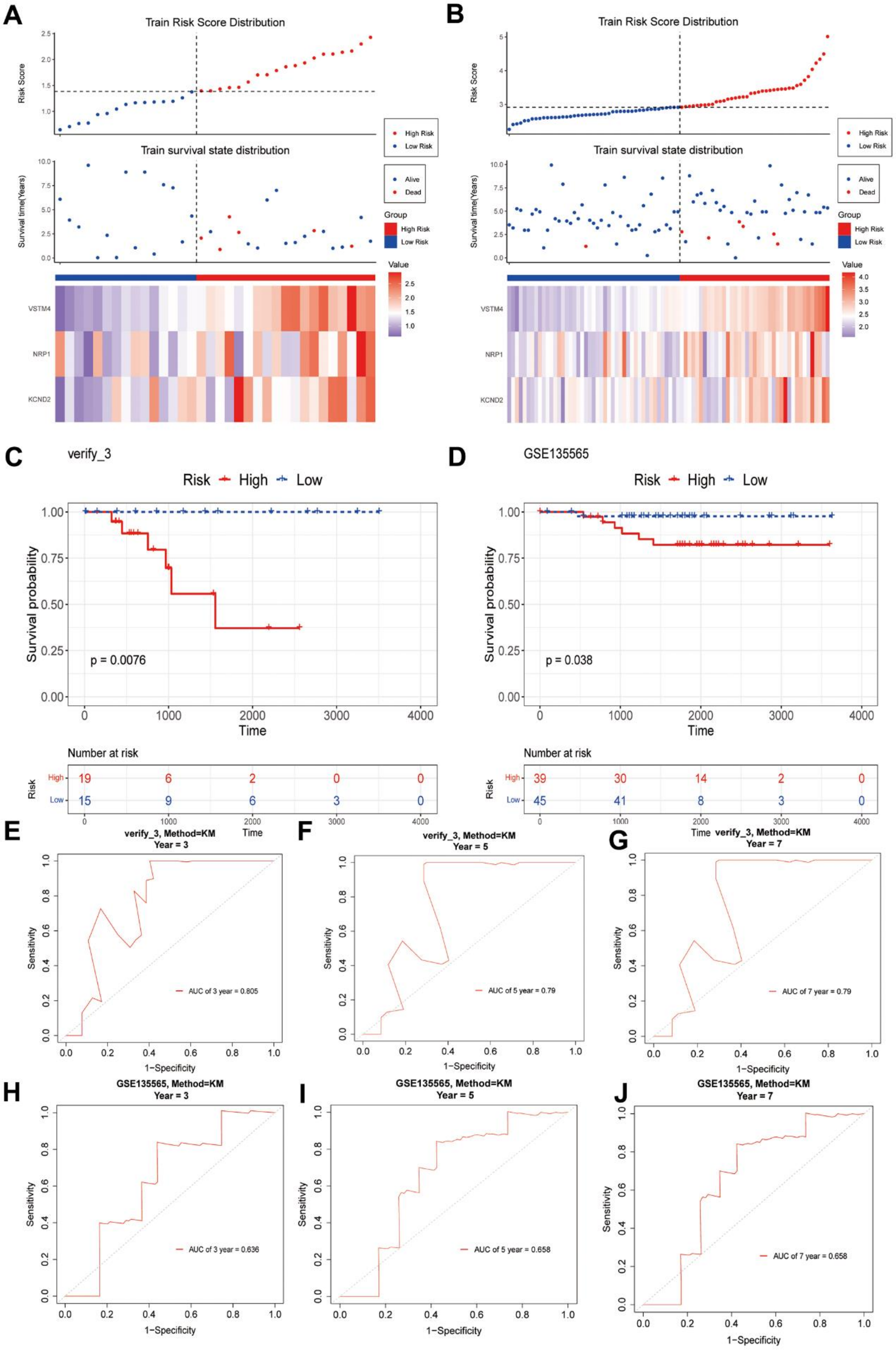

Supplementary Figure 2. The risk score was validated in three internal validation groups and one external validation group GSE135565. (**A**–**J**) The results of both internal and external validations were similar to those of the training set, which confirmed the good predictive performance of the risk model.

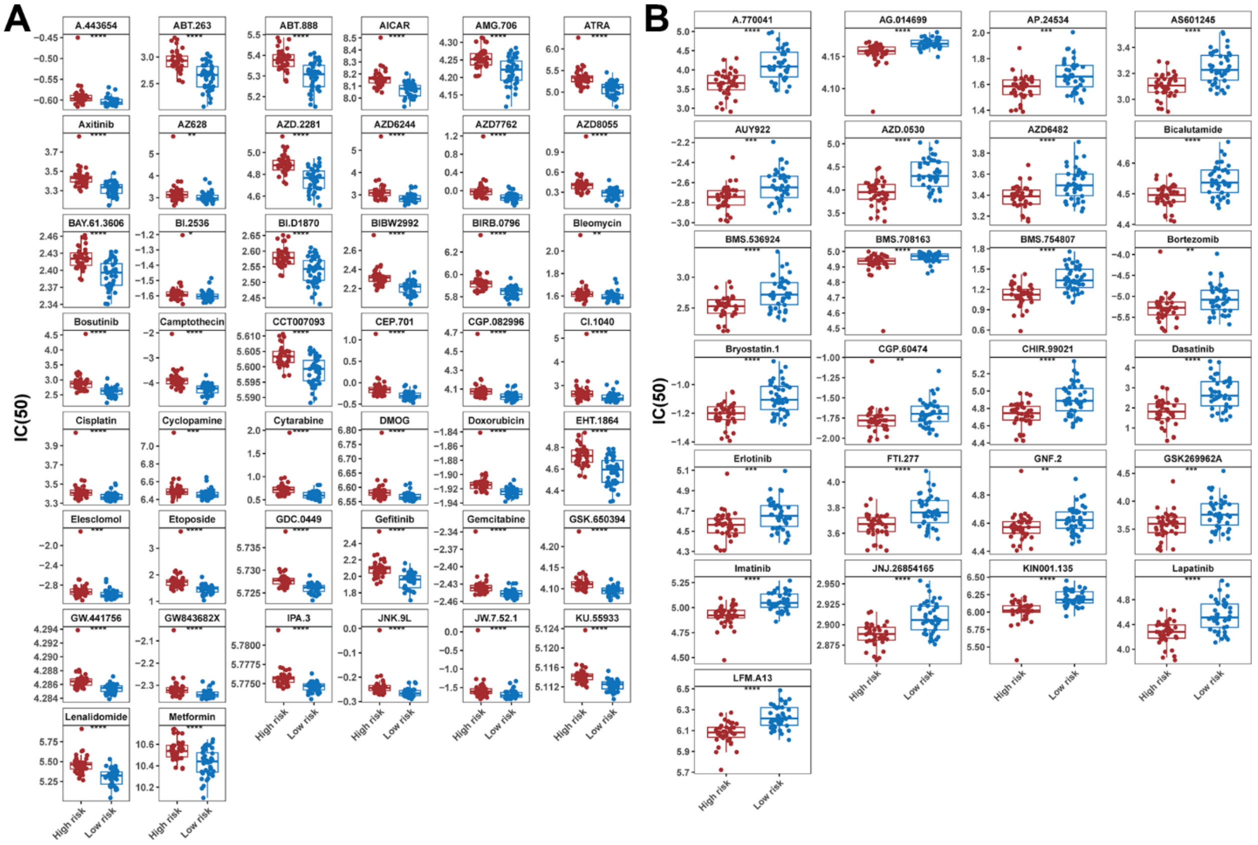

Supplementary Figure 3. Drug sensitivity in the high-risk and low-risk groups. Comparison of the IC_{50} for 138 drugs between the high-risk and low-risk groups in the training set. A total of 69 drugs were identified with significant differences between the high-risk and low-risk groups: (**A**) Drugs with lower IC_{50} in the high-risk group compared to that in the low-risk group. (**B**) Drugs with higher IC_{50} in the high-risk group compared to those in the low-risk group.

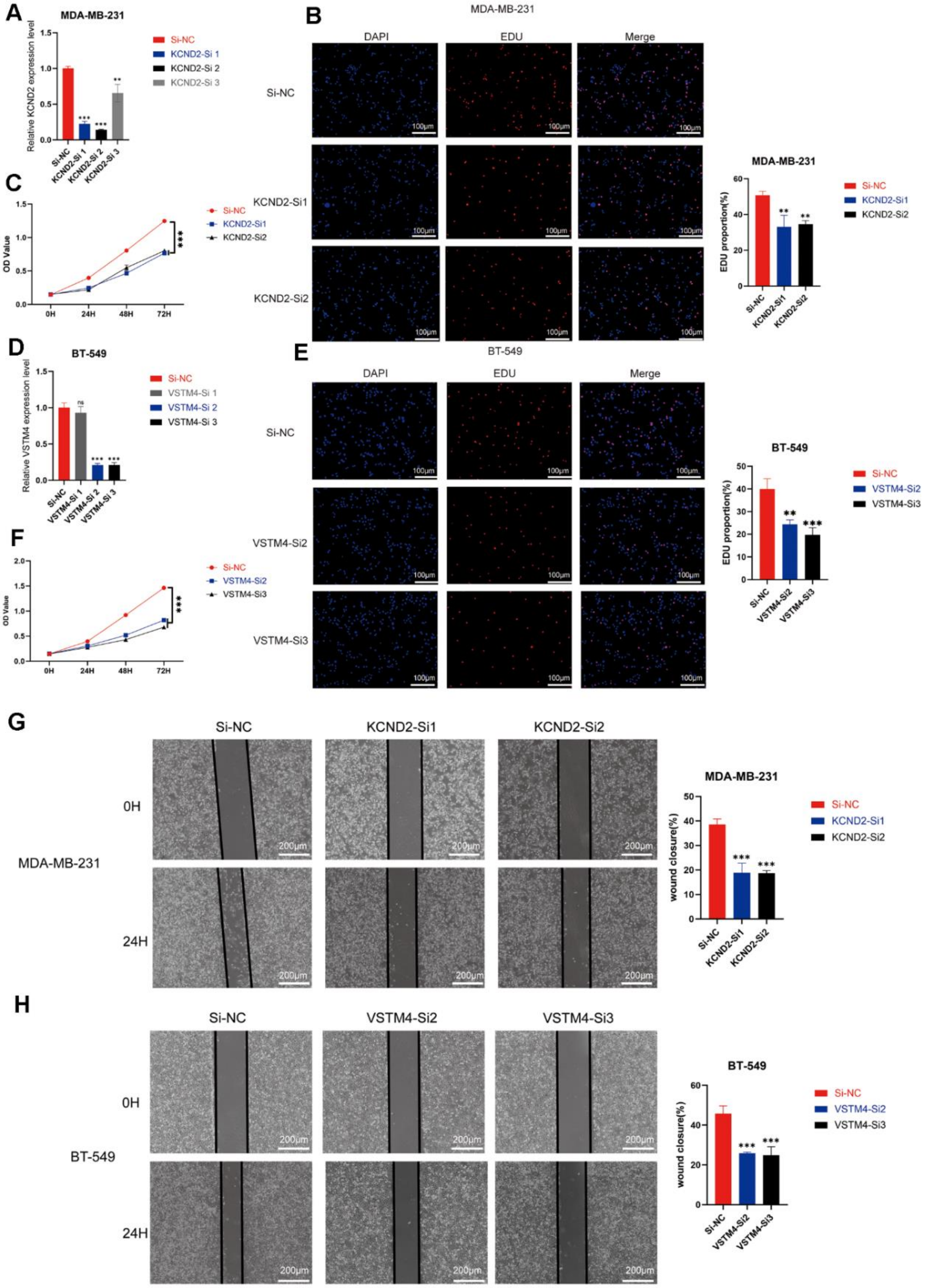

Supplementary Figure 4. Depletion of KCND2 and VSTM4 reduces TNBC cell proliferation and migration *in vitro*. (**A**) The knockdown efficiency of KCND2 siRNA in MDA-MB-231 cells was validated. (**B**) EdU incorporation analysis and (**C**) CCK-8 assay demonstrated that the knockdown of KCND2 affects cell proliferation in MDA-MB-231 cells. (**D**) The knockdown efficiency of VSTM4 siRNA in BT-549 cells was verified. (**E**) EdU incorporation analysis and (**F**) CCK-8 assay indicated that the knockdown of VSTM4 affected the cell proliferation in BT-549 cells. (**G**) The wound healing assay revealed that the knockdown of KCND2 impacted MDA-MB-231 cell migration. (**H**) Wound healing assay results showed that the knockdown of VSTM4 affected BT-549 cell migration. NS, no significance, *P < 0.05, **P < 0.01, ***P < 0.001.
