# Supplementary material for: Constructing a novel prognostic model for triple-negative breast cancer based on genes associated with vasculogenic mimicry
Source: Aging (Albany NY). 2024 May 8;16(9):8086–109. doi: 10.18632/aging.205806 (PMC11132006; doi:10.18632/aging.205806)
Supplement: Supplementary Table 1 [file aging-16-205806-s002.pdf]

SUPPLEMENTARY TABLE

Supplementary Table 1. 24 VM.

ID
TFPI
SERPINF1
TF
MAPK1
PIK3CA
ROCK1
VEGFA
NOTCH1
ROCK2
MAPK3
EPHA2
LAMC2
CDH5
KDR
PTGS2
MMP9
SNAI1
TWIST1
MMP2
LOXL2
TFPI2
SNAI2
TGFB1
TWIST2
